# Targeted delivery of siRNA against sodium-dependent phosphate co-transporter 2b (NPT2b) via functionalized mesoporous silica nanocarriers suppresses breast cancer cell growth

**DOI:** 10.1016/j.jgeb.2026.100825

**Published:** 2026-09-30

**Authors:** Fatemeh Yazdi Samadi, Morteza Farab, Mahdieh Emadi, Marzieh Rad Niknam, Maryam Yousefi, Arash Minai-Tehrani

**Affiliations:** a Nanobiotechnology Research Center, Avicenna Research Institute, ACECR, Tehran, Iran

**Keywords:** NPT2b, siRNA, Mesoporous silica nanoparticle, Breast cancer cells

## Abstract

The sodium-dependent phosphate co-transporter 2b (NPT2b) plays an important role in maintaining phosphate homeostasis. In previous studies, we reported that high dietary inorganic phosphate consumption could increase NPT2b expression in mice while knockdown of NPT2b expression has been shown to reduce tumorigenesis. NPT2b has been found to be highly expressed in human breast cancer tissues. The correlation between elevated NPT2b expression in the breast tissue and poor prognosis in oncogenic breast diseases led us to investigate whether knockdown of NPT2b could regulate breast cancer growth. To address this issue, breast cancer cells were transfected by small interfering RNA (siRNA) directed against NPT2b (siNPT2b), complexed with a mesoporous silica nanoparticle (MSN) grafted with polyethyleneimine (PEI) and modified with folic acid as a nonviral vector (hereafter, functionalized MSN). We used two distinct PEI molecular weights (10 and 25 kDa) to optimize the balance between nucleic acid delivery efficiency and cytotoxicity of cationic modified MSN. In this study, we developed an effective nanocarrier to deliver siRNA into breast cancer cells. Our findings demonstrated that functionalized MSN effectively targeted the folate receptor and delivered siNPT2b, resulting in the downregulation of NPT2b expression, possibly through regulation of phosphate consumption. Our results strongly indicate that siNPT2b delivery through our engineered nanocarrier especially when grafted with 10 kDa PEI can influence breast cancer growth and offers valuable insights into the development of nonviral nanocarriers for siRNA delivery, potentially contributing to the advancement of targeted cancer therapy.

## 1 Introduction

Breast cancer stands as the primary cause of cancer-related deaths among women globally. Despite advancements in its treatment, it still poses significant challenges, with the five-year survival rate showing minimal improvement over time. Traditional approaches like radiotherapy and chemotherapy, while commonly used, often come with significant drawbacks and limitations.^1^, ^2^, ^3^, ^4^ In recent times, therapeutic nucleic acid delivery has been proposed as a promising tool to enhance treatment efficacy and mitigate side effects.^5^ It is therefore important to develop a targeted gene delivery system and determine novel target molecules to enhance the efficacy of breast cancer treatments.

Inorganic phosphate holds pivotal roles in numerous biological processes and signaling pathways. Its uptake is facilitated by three families of sodium-dependent phosphate co-transporters (NPT1, NPT2, and NPT3), each comprising multiple members. Within these families, NPT2 is considered to be crucial for maintaining phosphorus homeostasis in mammals. Among its members, NPT2b, characterized by multiple transmembrane domains, regulates the consumption of transcellular phosphate, thereby contributing to phosphate homeostasis and tumorigenesis.^6^, ^7^, ^8^, ^9^, ^10^ Human breast tissue exhibits both protein and mRNA expression of NPT2b which is upregulated in the majority of breast cancer tumor samples.^8^, ^11^ Altered NPT2b expression has been documented in multiple malignancies.^12^, ^13^, ^14^ Therefore, recent findings have identified NPT2b as a potential molecular marker across diverse cancer types. However, existing evidence regarding its regulatory function and its impact on clinical outcomes in breast cancer remains limited, highlighting the need for further investigation.

Inorganic nanoparticles have been widely applied in various biological applications. Among them, Mesoporous Silica Nanoparticles (MSNs) are silica-based materials characterized by large surface areas, distinctive porous structures, substantial pore volumes, and uniformly adjustable pore sizes. These unique features have attracted significant attention in recent years for their potential in biomedical applications.^15^, ^16^, ^17^, ^18^, ^19^, ^20^, ^21^, ^22^, ^23^ Based on these, we developed a type of MSN-based system for oligonucleotide delivery, which is grafted with PEI and functionalized by folic acid. These vectors could initiate RNAi in breast cancer cells successfully. Complexing with PEI as a polymer for grafting has been reported to facilitate the cargo uptake into the cells and regulate endosomal escape for effective oligonucleotide delivery.^24^, ^25^, ^26^, ^27^, ^28^ Over the use of two distinct PEI molecular weights (10 and 25 kDa), it was possible to identify a carrier that maintained high cellular uptake and transfection efficacy with minimal cytotoxicity.

Although MSN/PEI platforms have been described for gene delivery, high molecular weight PEI (e.g., 25 kDa) causes marked cationic toxicity, limiting its translation. In addition, using targeted nanocarriers to silence inorganic phosphate transporters (specifically NPT2b) remains an unexplored therapeutic strategy in breast cancer. Here, we addressed both challenges by: (i) suggesting NPT2b-mediated phosphate uptake as a targetable vulnerability in breast cancer, and (ii) adjusting the MSN modification using lower molecular weight PEI (10 kDa vs. 25 kDa) to preserve delivery efficiency while minimizing cytotoxicity.

Hua et al. have reported that a high dietary phosphate intake elevated NPT2b expression, thereby promoting tumorigenesis and protein translation.^29^ Conversely, our prior research demonstrated that siRNA targeting NPT2b could impede lung cancer growth, diminish cancer cell proliferation while promoting apoptosis.^30^ Ye et al. observed significant upregulation of NPT2b in bladder cancer cell lines and clinical samples. Enhanced NPT2b expression has been reported to correlate with larger tumor size, while its reduction in bladder cancer cell lines inhibited cellular viability and suppressed xenograft tumor growth in vivo.^31^ These results prompt the crucial inquiry of whether modulating cellular phosphate transport in breast cancer cells through NPT2b knockdown could yield therapeutic benefits. To address this issue, we employed siRNA complexed with MSNs grafted using PEI (10 kDa) and functionalized with folic acid as a nonviral vector to silence NPT2b expression, and confirmed the consequences on cell proliferation in breast cancer cells.

## 2 Material and methods

### 2.1 Materials

Cetyltrimethylammonium bromide (CTAB), tetraethyl orthosilicate (TEOS), fluorescein isothiocyanate (FITC), folic acid (FA), 3-aminopropyltriethoxysilane (APTES), 3-glycidyloxypropyltrimethoxysilane (GPTMS), 1-ethyl-3-(3-dimethylaminopropyl)carbodiimide (EDC), N-hydroxysuccinimide (NHS), and branched polyethyleneimine (PEI; 10 and 25 kDa) were obtained from Sigma-Aldrich and Alfa Aesar. Diethyl ether, *N*,*N*-dimethylformamide (DMF), and dimethyl sulfoxide (DMSO) were obtained from Merck. Synthetic siRNA was purchased from Microsynth (Balgach, Switzerland). All infrared spectra were recorded using a Thermo Nicolet 5700 FTIR spectrometer (Waltham, MA, USA). Zeta potential measurements were performed using a Zetasizer Nano ZS (Malvern Instruments, UK). The presence of FITC incorporated into the silica shell was confirmed using an inverted fluorescence microscope (Olympus, Japan). This study was conducted in accordance with the ethical principles and national norms and standards for conducting medical research in Iran (Approval ID: IR.NIMAD.REC.1397.368), as evaluated by the Research Ethics Committees of the National Institute for Medical Research Development.

### 2.2 Synthesis of mesoporous silica nanoparticles

CTAB (1.0 g, 2.74 mmol) and NaOH (2.0 M, 3.50 mL) were added to deionized water (480 mL). Tetraethyl orthosilicate (5.0 mL, 21.9 mmol) was added dropwise at a rate of 1 μL min-1. The reaction mixture was stirred at 80 °C for 2 h resulting in the formation of white precipitates (as-made MSN). The reaction mixture was cooled to room temperature, and the precipitate collected by centrifugation at 10,000 rpm for 10 min. The precipitate was washed twice with deionized water and twice with methanol, and then dried in an oven at 85 °C overnight. To remove the surfactant template, the obtained MSNs were refluxed in a mixture of methanol (90 mL) and hydrochloric acid (12.1 M, 37% *w*/*v*, 5.0 mL) for 24 h. The product was then collected by centrifugation (10,956 ×*g*, 10 min) washed twice with water and twice with methanol, and dried again at 85 °C overnight.^32^

### 2.3 Synthesis of FITC-functionalized mesoporous silica nanoparticles (FITC-MSNs)

In order to prepare MSN attached to FITC, FITC-MSNs were first synthesized. FITC (5.5 mg, 0.014 mmol) was dissolved in 3 mL of absolute ethanol, and aminopropyltriethoxysilane (APTES; 12 μL, 0.05 mmol) was then added. The mixture was stirred at room temperature for 2 h in darkness to allow conjugation. Subsequently, tetraethyl orthosilicate (TEOS, 5.0 mL, 22.4 mmol) was added to the solution for another 2 h.

In a separate vessel, CTAB (1.00 g, 2.74 mmol) was dissolved in deionized water (480 mL) containing NaOH (2.0 M, 3.5 mL), and the mixture was heated to 80 °C under continuous stirring. The FITC/APTES/TEOS solution was added dropwise to the CTAB solution at a rate of 1 mL min^−1^. The reaction mixture was stirred at 80 °C for 2 h, resulting in the formation of FITC-labeled MSNs. After completion, the mixture was cooled to room temperature, and the product was collected by centrifugation at 10,956 ×*g* for 10 min. The solid was washed twice with deionized water and twice with methanol and then dried in an oven at 85 °C overnight. To remove the surfactant template, the dried product was refluxed in a mixture of methanol (90 mL) and hydrochloric acid (HCl, 12.1 M, 5.00 mL) for 24 h. The material was then collected by centrifugation, washed twice with water and twice with methanol, and dried again at 85 °C overnight.

### 2.4 Functionalization of MSNs with folic acid and polyethyleneimine

Folic acid was activated in the presence of EDC and NHS. Folic acid (0.26 g, 0.59 mmol) was dissolved in dimethyl sulfoxide (DMSO, 10 mL), followed by the addition of EDC (0.24 g, 1.25 mmol) and NHS (0.40 g, 3.48 mmol). The mixture was stirred at room temperature for 24 h in darkness to complete the activation process.

Separately, branched polyethyleneimine (PEI, 10 or 25 kDa; 0.15 g, corresponding to approximately 3.48 mmol of amine repeat units) and 3-glycidyloxypropyltrimethoxysilane (GPTMS, 1 mL, 4.5 mmol) were mixed in *N*,*N*-dimethylformamide (DMF, 50 mL) and stirred at 80 °C for 24 h. After this time, mesoporous silica nanoparticles (FITC-MSNs; 0.10 g) were added to the reaction mixture, and the suspension was stirred for an additional 24 h at 80 °C.

The resulting product was collected by centrifugation at 10,956 ×*g* for 10 min, washed with deionized water and ethanol, and dried by freeze-drying. The obtained FITC-MSN/PEI was then added to the activated folic acid solution and stirred on a roller mixer at room temperature for 4 h. The final product was washed with diethyl ether, resuspended in a portion of the original PEI solution prepared in isopropanol, and stirred for an additional 2 h.

The resulting folic acid- and PEI-functionalized FITC-MSNs were designated according to the molecular weight of PEI as MSN(FITC)@PEI 10 kDa/FA and MSN(FITC)@PEI 25 kDa/FA, hereafter referred to as “functionalized MSN (PEI 10)” and “functionalized MSN (PEI 25),” respectively. The products were isolated and stored for further use.

### 2.5 siRNA complexation, encapsulation, protection and release assay

For complexation, following calculation of the appropriate amounts of functionalized MSN to make different weight ratios with siRNA, the calculated amounts of siRNA were added drop by drop into a microtube containing the appropriate amount of modified MSN and the resulting solution was placed at the room temperature for 30 min. The solutions prepared with different weight ratios were evaluated to investigate the ability of modified MSN to bind to siRNA by 2% agarose gel. Electrophoresis was performed for 40 min with a voltage of 70 and after this time, the gel was observed with the aid of Gel documentation device. For Protection and release assay, prepared complexes and naked siRNA were separately incubated with RNase-I at 37 °C for 30 min. The reaction was inactivated by adding EDTA, and the complexes were subsequently treated with sodium dodecyl sulfate (SDS) in 0.1 M NaOH (pH 7.2) at final concentration of 1.0%. siRNA encapsulated in nanoparticles was assessed using Eppendorf BioPhotometer. Briefly, siRNA MSNs were centrifuged for 30 min at 4 °C, and then siRNA concentration in the supernatant was determined. The encapsulation efficiency (EE%) was expressed as the percentage of the amount of siRNA encapsulated in the nanoparticles in relation to the total amount of siRNA initially added.

### 2.6 Hemolysis assay

The obtained RBCs were washed three times with sterile isotonic solution. The RBC suspension was mixed with different concentrations of functionalized MSN (PEI 10). Here, SDS was used as the positive control. All samples were incubated at 37 °C for 1 h., followed by centrifugation. The absorbance of the supernatant was evaluated by Eppendorf BioPhotometer.

### 2.7 Transfection, reverse transcription PCR and qRT-PCR

MCF-7 and MDA-MB 231 cells were seeded in appropriate culture plates. On the day of transfection, siRNA against NPT2b or negative control siRNA (NC siRNA) were complexed with functionalized MSN (PEI 10) and then added to the cells. The transfected culture was incubated at 37 °C with 5% CO_2_ and 85% humidity. Then RNA was extracted from cells using Qiagen RNeasy Mini Kit according to manufacturer's instructions, and RNA concentration was quantified using Thermo Scientific NanoDrop™. Reverse transcription was performed using random hexamer primer and M-MLV Reverse Transcriptase (Roche). The cDNA was then amplified by PCR using the specific primers for NPT2B and GAPDH. The PCR products were then analyzed on agarose gel and the bands were visualized. For qRT-PCR, RNA was extracted and cDNA was synthesized as described above. qRT-PCR was performed using specific primers for the target gene and housekeeping gene in the presence of SYBR Green master mix on a Rotor-Gene Q real-time PCR. Gene expression levels were analyzed relative to the housekeeping gene.

### 2.8 Viability test

MTT assay was performed as described elsewhere.^33^ Briefly, MCF-7, MDA-MB 231 (breast cancer cells) and MCF 10 A (normal breast epithelial cell) cell were seeded in 96 well plate and incubated with the medium containing different concentrations of functionalized MSN (prepared by PEI 10 kDa or 25 kDa) (concentrations of 10, 25, 50, and 75 μg mL^−1^) in a constant temperature incubator for 48 h. The absorbance of each well following adding MTT solution and then DMSO was measured by a micro plate reader (Thermo scientific, USA) and the viability rate was calculated. The Same producer was performed to examine the viability of prepared complexes after 48 h. Briefly, MCF-7 and MDA-MB 231 cells were incubated with the medium containing complexes of siRNA with functionalized MSN (PEI 10 kDa) in a constant temperature incubator. The absorbance of each well following adding MTT solution and then DMSO was measured by a micro plate reader and then the viability rate was calculated.

### 2.9 Flow cytometric analysis of folate receptor-mediated cell association of FITC-conjugated MSNs

MCF-7 cells were incubated with FITC-conjugated nanocarriers for 30 min. After detaching and washing, the cells were analyzed using a Partec PAS II flow cytometer equipped with two lasers (488 nm and 620 nm) and four-color detection. Untreated MCF-7 cells were used as the negative control to define the main cell population and fluorescence baseline. First, the control cells were gated on the FSC versus SSC dot plot to exclude debris and non-cellular events. The gated cell population was then displayed on a FITC (FL1) versus SSC plot. The fluorescence positivity threshold was determined according to the distribution of the control sample, and the positive region was defined at the end of the first logarithmic fluorescence scale where FITC-positive events appeared. FlowJo software was used for data acquisition and analysis.^34^ Cell-associated FITC was analyzed and reported as the percentage of FITC-positive cells.

### 2.10 Apoptosis assay

Apoptosis was evaluated using an Annexin V-FITC/Propidium Iodide (PI) double staining assay followed by flow cytometry. Briefly, cells were treated with the prepared functionalized MSN–siRNA complexes as mentioned earlier. After 48 h, the cells were harvested, washed and stained with Annexin V-FITC and PI. The stained cells were subsequently analyzed using a Partec PAS II flow cytometer. Cells were classified into viable, early apoptotic, late apoptotic, and necrotic populations based on their Annexin V and PI staining profiles. The percentage of cells in each population was determined to assess the apoptosis inducing effect of the prepared complexes.

### 2.11 Statistical analysis

Data were analyzed using GraphPad Prism version 8. Statistical significance was evaluated using one-way or two-way ANOVA, followed by Dunnett's comparison test where appropriate. *p* < 0.05, *p* < 0.01, *p* < 0.001 and *p* < 0.0001 were represented as *, **, *** and ****, respectively.

## 3 Results

### 3.1 Preparation of nanocarriers

Fig. 1, Fig. 2 illustrate the stepwise synthesis approach employed in this study. In the first step (Fig. 1a), the epoxy groups of 3-glycidyloxypropyltrimethoxysilane (GPTMS) undergo nucleophilic attack by primary amine groups in polyethyleneimine, resulting in the formation of GPTMS-PEI conjugates^35^, ^36^, ^37^. In the second step (Fig. 1b), mesoporous silica nanoparticles (MSNs) react with GPTMS-PEI, yielding MSN-PEI. In the third step (Fig. 1c), MSN-PEI is functionalized with folic acid activated by EDC/NHS chemistry, leading to the formation of MSN-PEI-FA. The schematic (Fig. 1d) depicts the interaction between MSN-PEI-FA and siRNA.

**Fig. 1:**
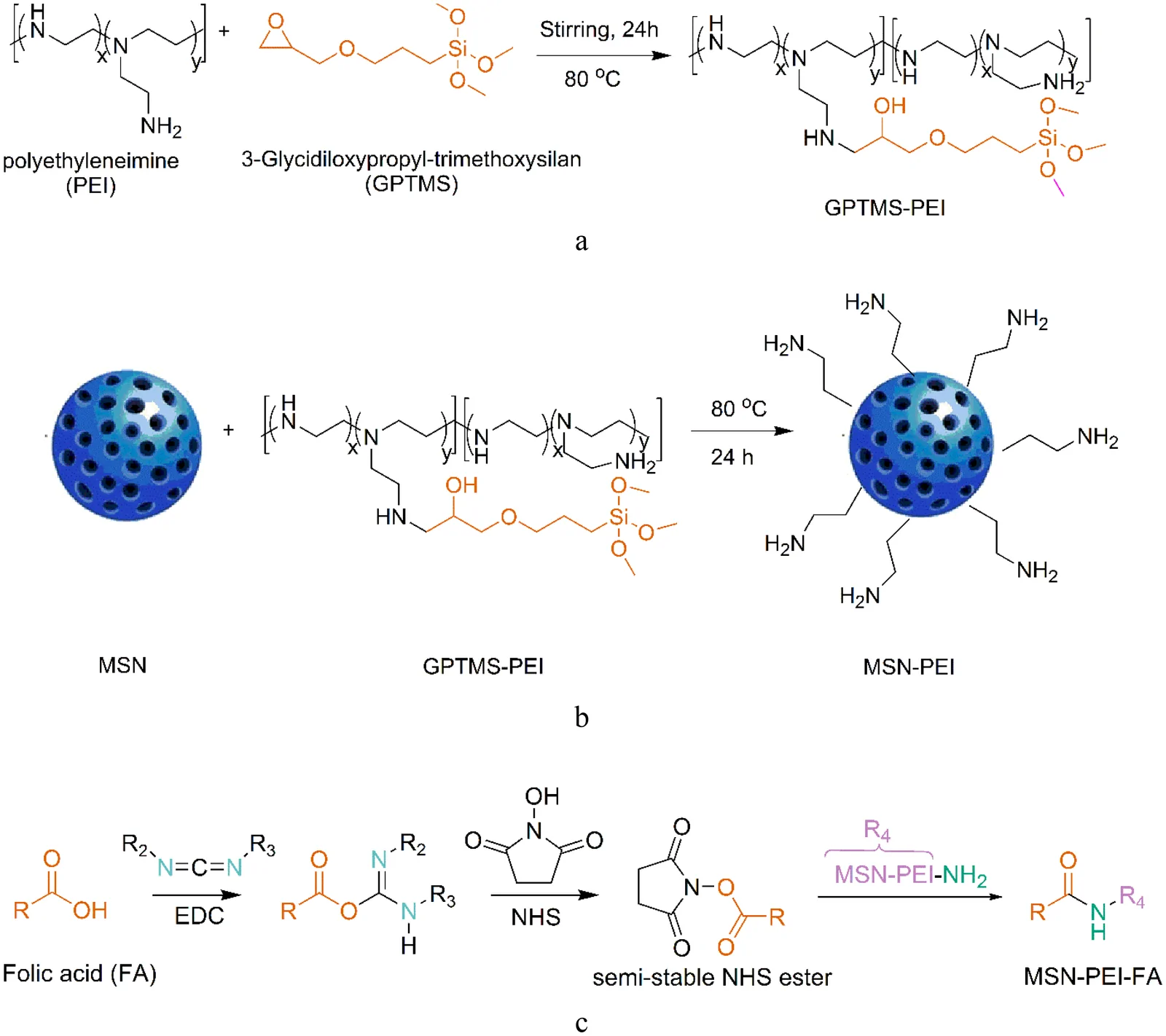

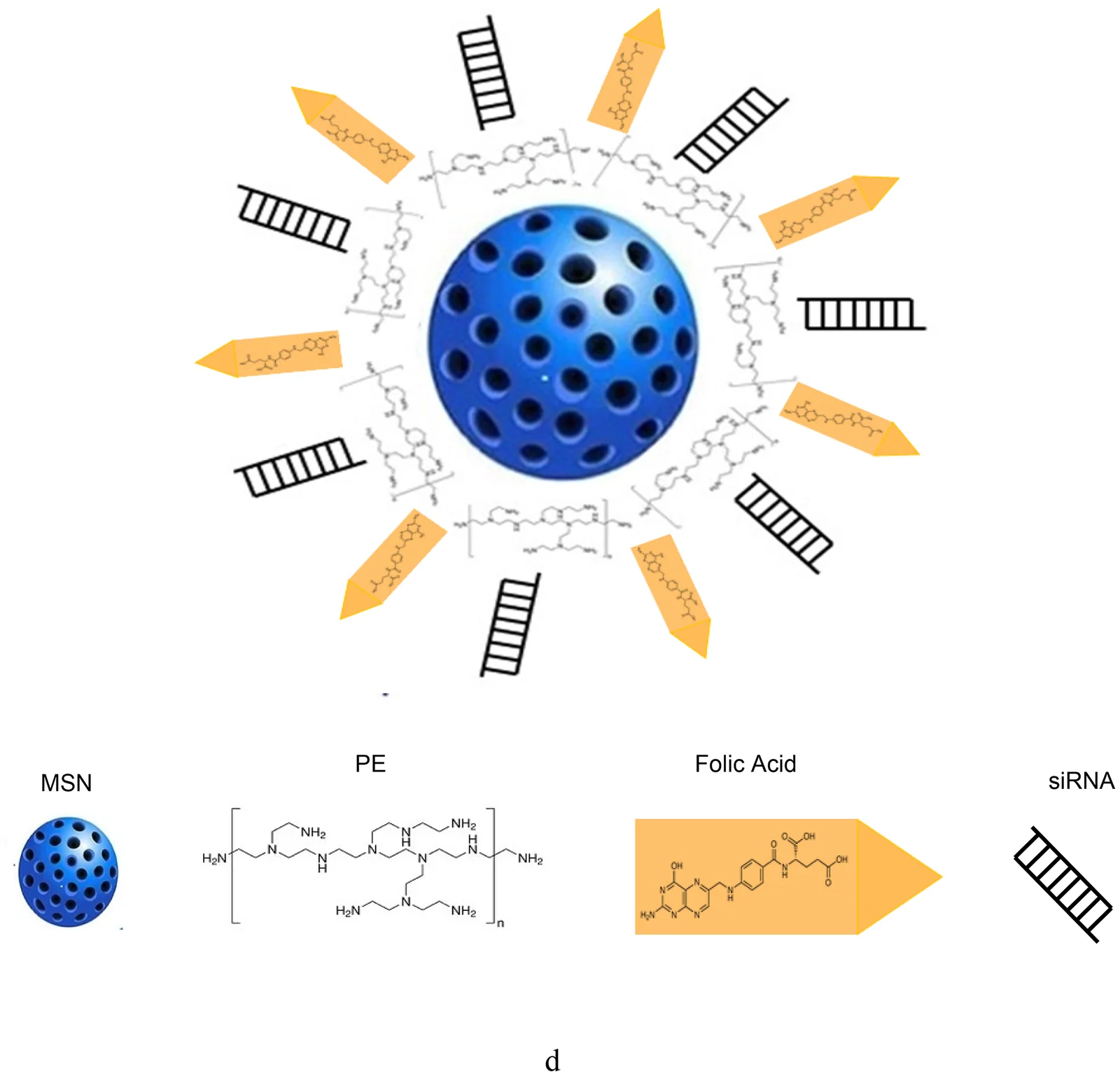
(a) Synthesis of GPTMS-PEI. (b) Synthesis of MSN-PEI (c) Synthesis of MSN-PEI-FA (d) The interaction between MSN-PEI-FA and siRNA. Synthetic method of preparing nanocarriers.

**Fig. 2:**
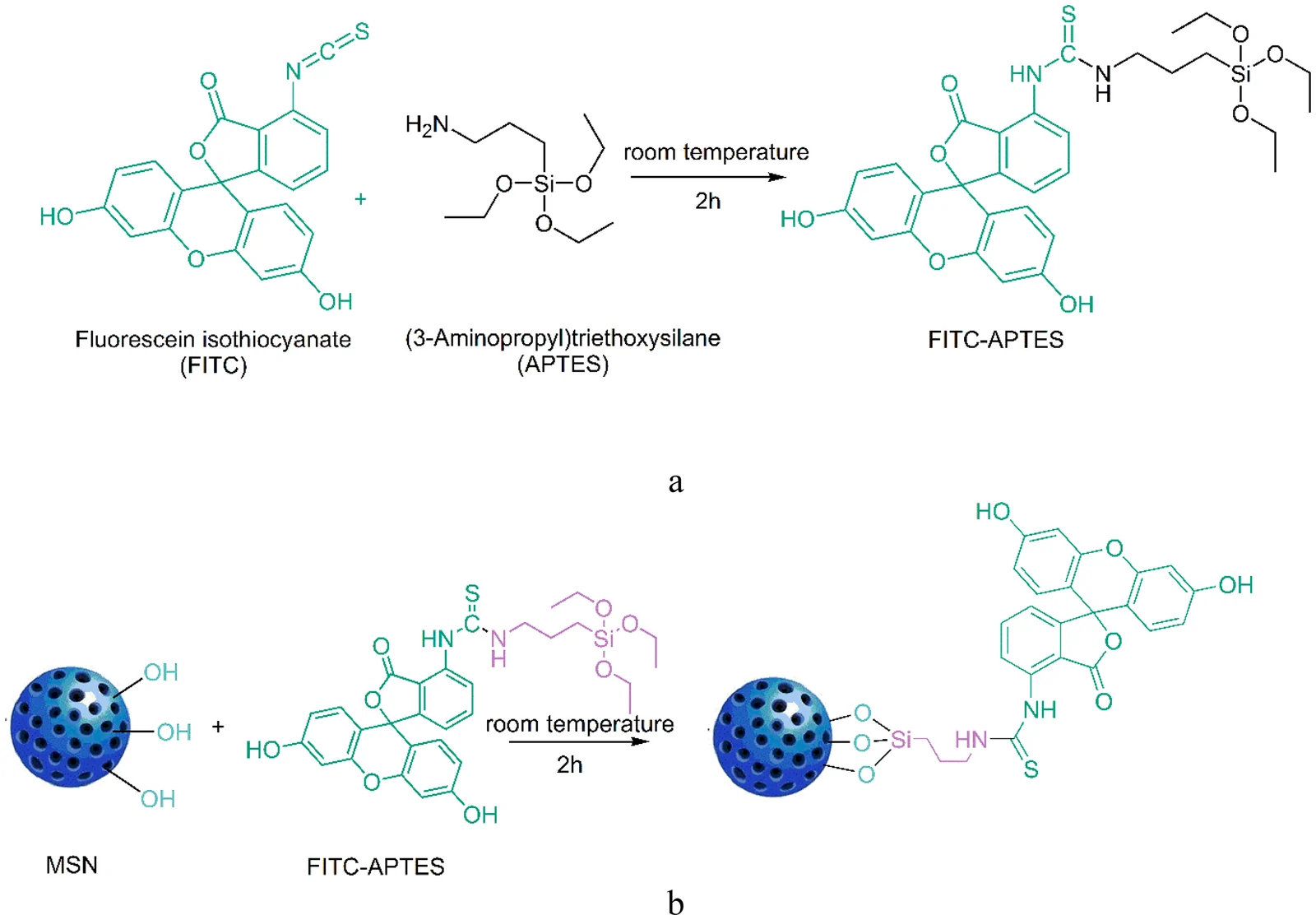
(a) The reaction between FITC and APTES (b) The reaction between FITC-APTES and MSN nanoparticles.

The MSN nanoparticles used to form the MSN-PEI-FA complex were all pre-conjugated with FITC using APTES in the reaction as shown in Fig. 2. The isothiocyanate group of FITC reacts with the amine groups of APTES (Fig. 2a) and then this complex reacts with the OH groups of the MSN surface (Fig. 2b), the addition of FITC during the synthesis process also causes simultaneous co-encapsulation of FITC dye molecules inside the porous structure of MSN.

### 3.2 Characterization of synthesized nanoparticles by SEM and TEM

Fig. 3a represents the scanning electron microscopy (SEM) images of the synthesized MSN particles. Due to the small pore size of MSNs (approximately 1–5 nm), the internal mesoporous structure is not visible under SEM. However, the images clearly reveal that the nanoparticles exhibit a well-dispersed, spherical morphology with uniform size distribution and an average particle diameter of approximately 81.9 nm. Transmission electron microscopy (TEM) analysis confirms the successful formation of a highly ordered mesoporous structure within the MSNs. High-resolution micrographs clearly show parallel 1D cylindrical channels and uniform hexagonal pore packing, which aligns well with the type IV isotherm profiles obtained from nitrogen adsorption-desorption measurements (Fig. 3b).

**Fig. 3:**
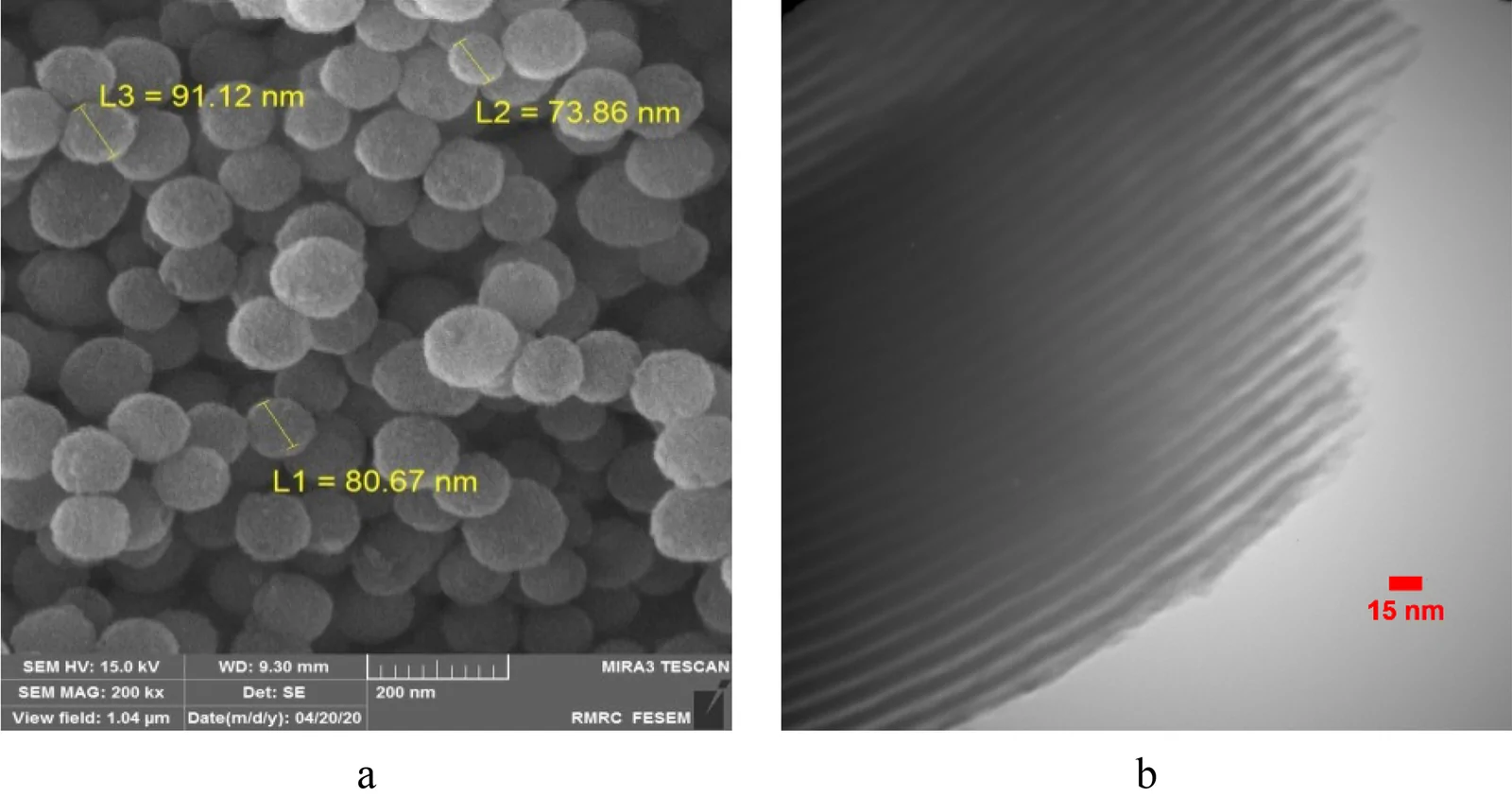
(a) SEM and (b) TEM images of synthesized MSN particles.

### 3.3 Characterization of nanocarriers by fluorescence microscopy

To confirm FITC attachment on the surface of MSNs, we evaluated the presence of fluorescence using fluorescence microscopy. Our results clearly demonstrated that functionalized MSN (PEI 25) (Fig. 4 left panel) and functionalized MSN (PEI 10) (Fig. 4 right panel) were uniformly dispersed while exhibiting clear and distinct fluorescence. These indicate that the PEI-FA modification on FITC-MSN does not significantly affect its fluorescence intensity.

**Fig. 4:**
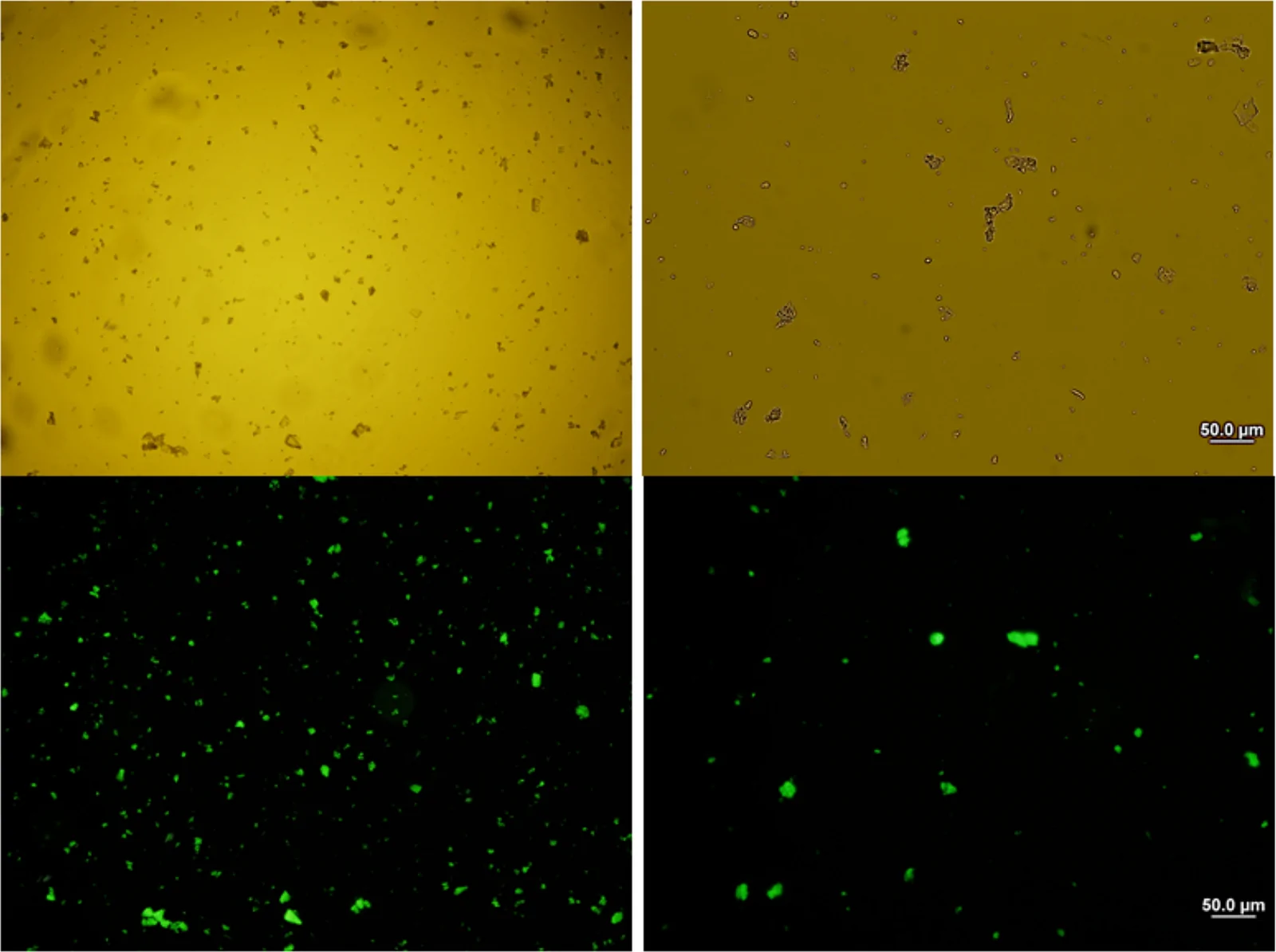
Microscopic images for aqueous suspension of functionalized MSN (PEI 25) (Left panel). Microscopic images for aqueous suspension of functionalized MSN (PEI 10) (Right panel).

### 3.4 Nitrogen adsorption characterization

The textural properties of the synthesized MSN and its functionalized derivatives were evaluated by nitrogen (N₂) adsorption–desorption analysis. The corresponding isotherm is presented in Fig. 5. All samples displayed adsorption–desorption behavior characteristic of mesoporous silica, with a pronounced increase in N₂ uptake at high relative pressures and a hysteresis between the adsorption and desorption branches. These features are consistent with a mesoporous structure according to the IUPAC classification.^38^ The changes observed in the isotherm profiles following functionalization indicate modifications of the accessible pore structure while maintaining the overall mesoporous character of the silica framework. The BET specific surface area, total pore volume, and mean pore diameter obtained from the N₂ sorption measurements are summarized in Table 1. Unmodified MSN exhibited a high specific surface area of 626.4 m^2^ g^−1^, a total pore volume of 0.4756 cm^3^ g^−1^, and a mean pore diameter of 3.04 nm, consistent with the typical textural characteristics of mesoporous silica nanoparticles.^39^ Following FITC incorporation, the specific surface area and total pore volume decreased markedly to 94.7 m^2^ g^−1^ and 0.319 cm^3^ g^−1^, respectively. Subsequent PEI functionalization resulted in a further decrease in the accessible surface area and pore volume. The BET surface area decreased to 42.9 m^2^ g^−1^ for MSN-FITC-PEI 25 kDa and 21.2 m^2^ g^−1^ for MSN-FITC-PEI 10 kDa, while the corresponding pore volumes decreased to 0.2497 and 0.1267 cm^3^ g^−1^, respectively.

**Fig. 5:**
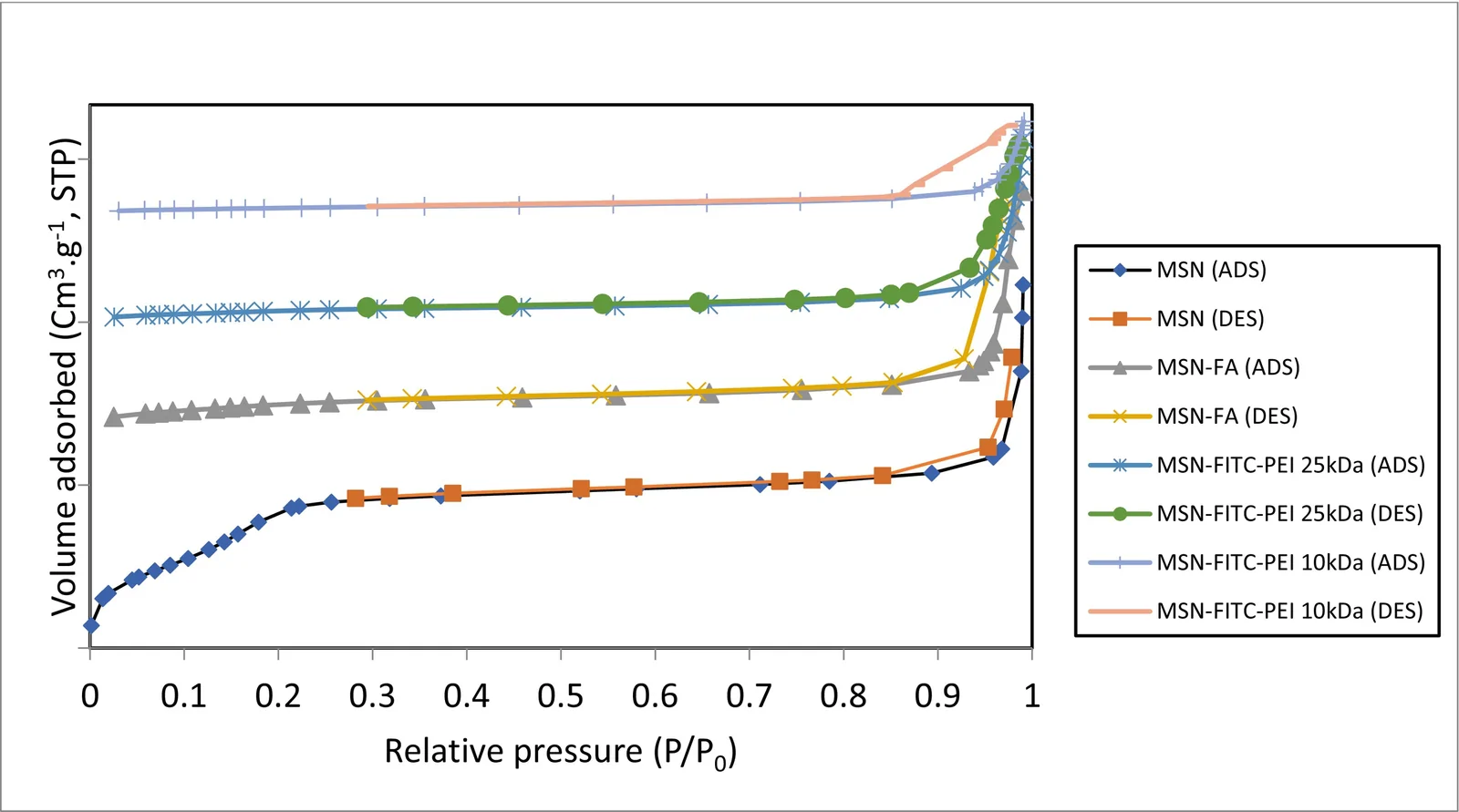
Adsorption (ADS)/desorption (DES) isotherms of MSN, MSN–FITC, MSN–FITC–PEI (25 kDa), and MSN–FITC–PEI (25 kDa)–FA.

**Table 1:** Surface area, pore volume, and pore diameter of MSNs.

Sample	Total pore volume (cm^3^g^−1^)	Mean pore diameter (nm)	specific surface area in (cm^3^g^−^^1^)
MSN	0.47	3.03	626.4
MSN-FITC	0.31	13.47	94.7
MSN-FITC-PEI 25 kDa	0.25	23.28	42.9
MSN-FITC-PEI 10 kDa	0.13	23.87	21.2

The progressive reduction in surface area and pore volume following functionalization is consistent with the incorporation of organic components into the MSN pore network. FITC incorporation and subsequent PEI grafting may partially occupy the pore space and restrict access to the internal pore surfaces, thereby reducing the amount of N₂ accessible during the physisorption measurement. Similar decreases in the textural parameters of mesoporous silica following surface functionalization or polymer grafting have been reported and are generally attributed to partial pore filling or pore blocking.^40^

### 3.5 FTIR of products

The FT-IR spectra of MSN, MSN–FITC, MSN–FITC–PEI (25 kDa), and MSN–FITC–PEI (25 kDa)–FA are presented in Fig. 6 (a-d). As shown in Fig. 6a, MSN exhibit a strong absorption band at 1047 cm^−1^, corresponding to the asymmetric stretching vibration of Si–O–Si, while the band at 801 cm^−1^ is attributed to the symmetric stretching vibration of Si–O–Si. The broad absorption band observed in the region of 3300–3400 cm^−1^ is assigned to the stretching vibrations of surface O—H groups, originating from silanol groups and adsorbed moisture.^41^, ^42^

**Fig. 6:**
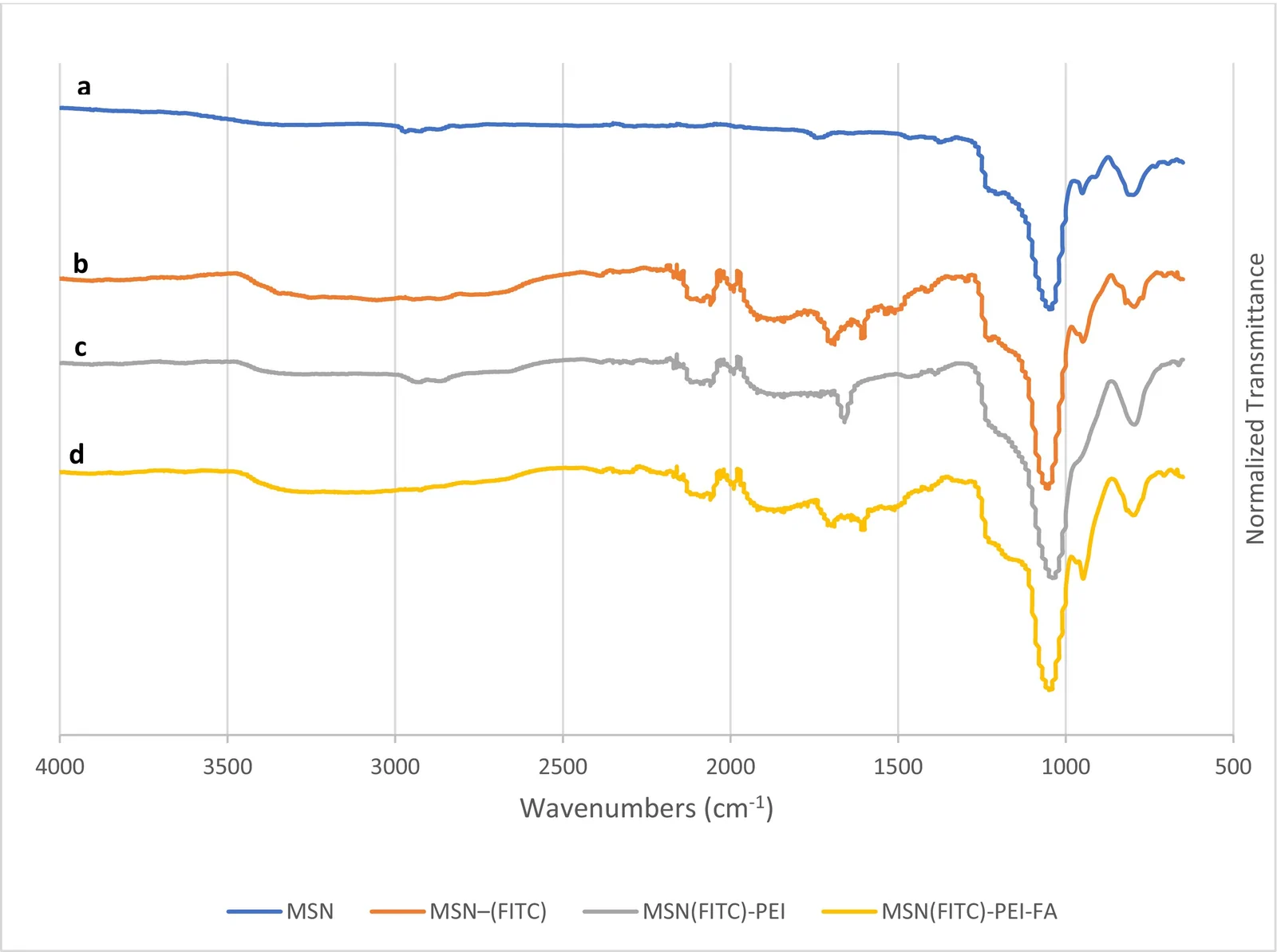
ATR-FTIR spectra of (a) MSN, (b) MSN (FITC), (c) MSN (FITC)-PEI (25 kDa) (d) MSN (FITC)-PEI (25 kDa)-FA. All spectra were min-max normalized to the primary Si–O–Si (1080 cm^−1^) band.

Following FITC conjugation (Fig. 6b), the characteristic bands of the silica framework are preserved, indicating that the mesoporous structure remains intact. In addition, new absorption bands appear after FITC modification, confirming the introduction of organic moieties onto the MSN surface. The bands at 2920 and 2850 cm^−1^ are attributed to C—H stretching vibrations of aliphatic groups from FITC and the organic linker. The absorption bands in the 1500–1600 cm^−1^ region are assigned to aromatic C=C stretching vibrations of the fluorescein ring system, partially overlapping with thiourea-related vibrations formed upon reaction of FITC with surface amine groups. Moreover, the band observed at 1630–1650 cm^−1^ is associated with C=O stretching and/or thiourea vibrations, providing evidence for the covalent attachment of FITC. Notably, the characteristic –N=C=S stretching vibration of free FITC (2100–2140 cm^−1^) is absent, indicating successful reaction of the isothiocyanate group with surface amines. A reduction in the intensity of the Si–OH band (950–970 cm^−1^) further suggests effective surface functionalization.

As shown in Fig. 6c, further modification with PEI (25 kDa) leads to additional spectral changes. The broad band in the 3200–3500 cm^−1^ region becomes more pronounced and is attributed to overlapping N—H stretching vibrations of primary, secondary, and tertiary amine groups in PEI, together with hydrogen-bonded hydroxyl groups. The increased intensity of bands at 2850–2950 cm^−1^ corresponds to C—H stretching vibrations of the PEI backbone, confirming successful polymer coating. In the fingerprint region, bands observed at 1600–1650 cm^−1^ are attributed to N—H bending vibrations, overlapping with aromatic vibrations from FITC, while bands at 1450–1550 cm^−1^ are characteristic of C—N stretching and N—H bending modes of PEI.

After conjugation of folic acid via EDC/NHS chemistry (Fig. 6d), additional spectral features attributable to FA and newly formed amide bonds are observed. The broad 3200–3500 cm^−1^ band is further intensified due to overlapping N—H and O—H stretching vibrations from FA, residual PEI amines, and surface hydroxyl groups. A prominent change is evident in the 1600–1700 cm^−1^ region, where the band at 1650–1680 cm^−1^ is assigned to amide I (C=O stretching) vibrations, confirming the formation of amide bonds between the carboxyl groups of folic acid and PEI amine groups. Additional bands in the 1500–1600 cm^−1^ region correspond to amide II vibrations as well as aromatic C=C stretching from the pterin and benzoyl rings of folic acid. The bands at ∼400–1450 cm^−1^ are attributed to C—N stretching and O—H bending vibrations, providing further evidence of FA immobilization. The decreased intensity of the Si–OH band (950–970 cm^−1^) indicates effective surface coverage after multistep functionalization. The same absorption bands were obtained when PEI (10 kDa) was used (data not shown). Overall, the sequential appearance of FITC-, PEI-, and FA-related absorption bands, together with the preservation of the silica framework vibrations, confirmed the successful stepwise functionalization of MSN.

### 3.6 Analysis of MSNs properties

Changes in Zeta potentials clearly demonstrate the surface modifications of MSNs. The functionalization of MSN has been reported to cause the changes in zeta potential of MSN. The net surface charge of FITC–MSN has been indicated to be negative as reported elsewhere, whereas the charge was reversed in the functionalized MSNs, showing values of +33.0 and + 34.4 mV when prepared by PEI 10 and 25 kDa, respectively, as measured by Zetasizer. This indicates successful attachment of PEI-FA and suggests that modified MSN nanoparticles can electrostatically interact with negatively charged siRNA. The size of functionalized MSNs (PEI 10 kDa and PEI 25 kDa) was 121 and 126.2 nm respectively. In addition, the results of encapsulation efficiency (EE%) revealed that MSNs could effectively encapsulate siRNA, showing values of 89.8% and 88.6% when prepared by PEI 10 kDa and PEI 25 kDa, respectively.

### 3.7 Gel retardation and release assay of siRNA

The cationic charge density of functionalized MSNs can form complexes with siRNA by an ionic interaction. as shown in Fig. 7a, the condensation of siRNA with the functionalized MSNs (grafted using PEI 25 kDa) at various weight ratios, was examined using a gel retardation assay. siRNA migration on the agarose gel was retarded with increasing amounts of modified MSN. By reaching the weight ratio to 10 and above, the movements of siRNA in the gel were completely stopped and they remained in the gel wells, which indicate their complete complexation to functionalized MSN. The degradation of siRNA by serum nuclease enzyme is the first biological obstacle when naked siRNA enters the body without a carrier. Therefore, we evaluated the ability of modified MSN to protect siRNA from degradative enzymes such as RNase as shown in Fig. 7b. Our result showed that functionalized MSN effectively protected siRNA from RNase degradation, whereas the naked siRNA was completely degraded, suggesting that functionalized MSN can protect siRNA from enzymatic degradation. Similar results were obtained with the functionalized MSN (grafted using PEI 10 kDa). Our retardation assay clearly showed at weight ratio of 20 and greater than that the complexations were well occurred (Fig. 7c). It protected siRNA against RNase-I and could release siRNA molecules nearly intact from the complexes following treatment (Fig. 7d). These results suggest the complexes are able to release and deliver their cargo inside the target cells.

**Fig. 7:**
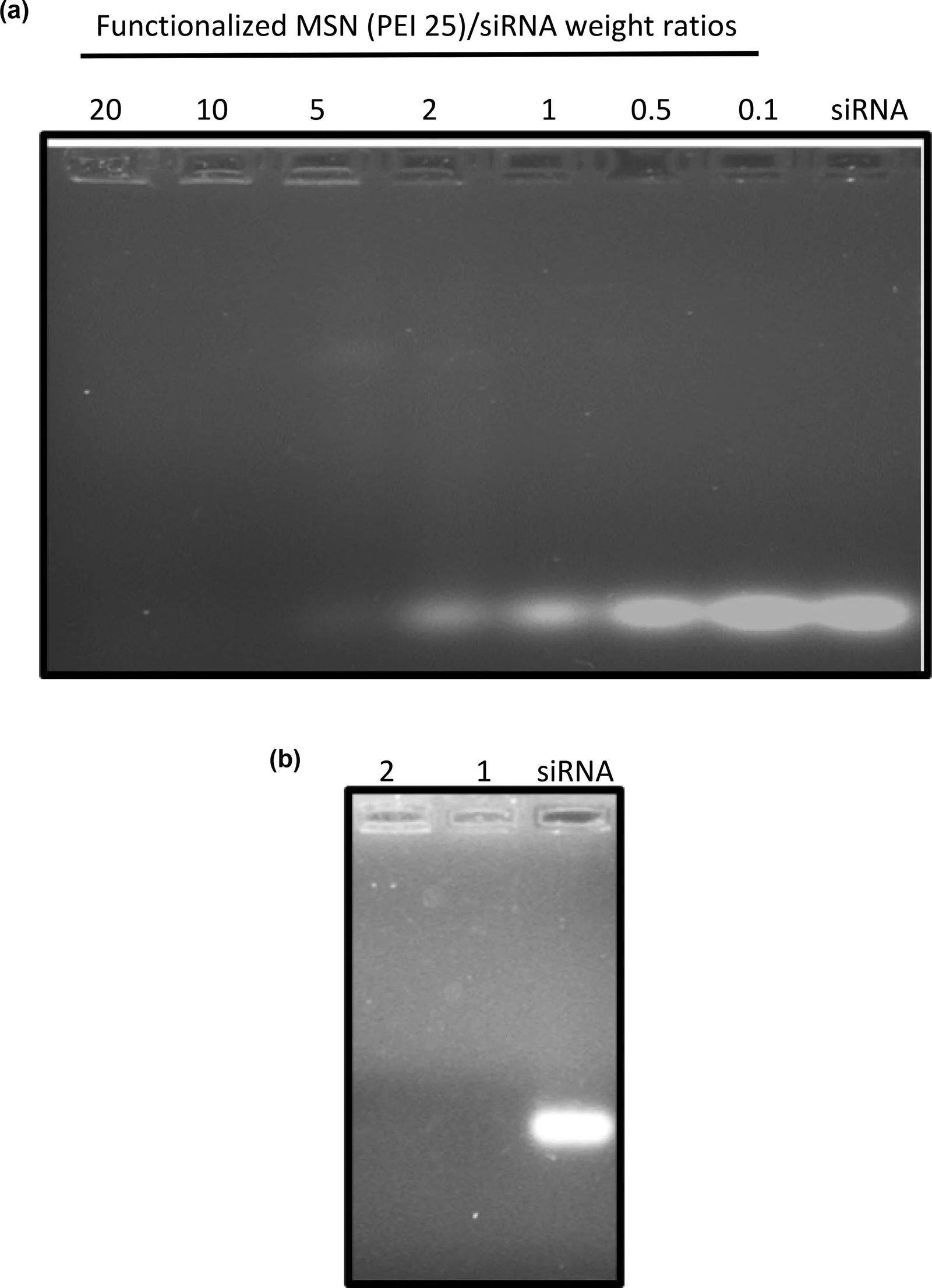

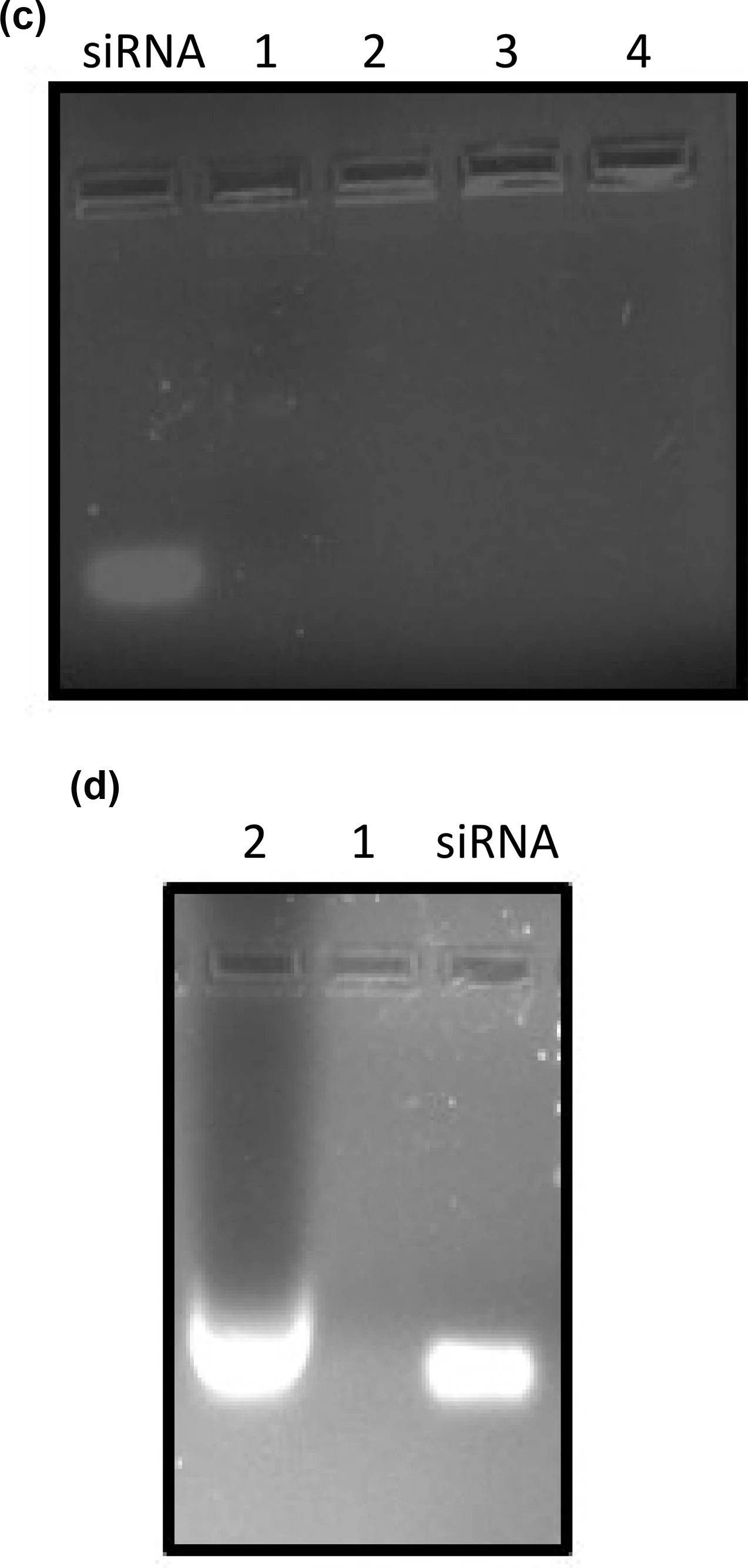
(a) Agarose gel electrophoresis was performed to evaluate the migration pattern of siRNA within the gel following forming complexes between functionalized MSN (PEI 25) and siRNA. 1: Naked siRNA, 2: Complex with weight ratio of 0.1, 3: Complex with weight ratio of 0.5, 4: Complex with weight ratio 1, 5: Complex with weight ratio 2, 6: Complex with weight ratio 5, 7: Complex with weight ratio 10, 8: Complex with weight ratio of 2 0. (b) Agarose gel electrophoresis was performed to evaluate the effect of RNase on the complexes. 1: Naked siRNA, 2: Naked siRNA in the presence of RNase, 3: functionalized MSN (PEI 25)/ siRNA complex with a weight ratio of 20 in the presence of RNase. (c) Agarose gel electrophoresis was performed to evaluate the migration pattern of siRNA within the gel following forming complexes between functionalized MSN (PEI 10) and siRNA. 1: Naked siRNA, 2: Complex with weight ratio of 15, 3: Complex with weight ratio of 20, 4: Complex with weight ratio 25, 5: Complex with weight ratio 30 (d) Agarose gel electrophoresis was performed to evaluate the release of siRNA following complexation. 1: Naked siRNA, 2: functionalized MSN (PEI 10)/ siRNA complex with a weight ratio of 25, 3: functionalized MSN (PEI 10)/ siRNA complex with a weight ratio of 25 following treatment with RNase and SDS with NaOH.

### 3.8 Comparing the cytotoxicity of MSNs with different PEI grafting

To evaluate the effects of different PEI grafting, we aimed to compare the cytotoxicity of engineered nanocarriers. Our results demonstrated that the functionalized MSN (PEI 25) exhibited toxic effects at higher concentrations, whereas the functionalized MSN (PEI 10) at the same concentrations was less cytotoxic to MCF-7, MCF-10 A (Fig. 8a) and MDA-MB-231 cells (Fig. 8b). According to the results shown in Fig. 8 a and b, the functionalized MSN (PEI 10) showed a similar pattern of cytotoxicity at the tested concentrations across the three tested cell lines, demonstrating acceptable cytocompatibility at the optimal concentrations. In contrast, the functionalized MSN (PEI 25) did not show such pattern and displayed a more pronounced concentration-dependent increase in cytotoxicity. This indicates that the toxicity of nanocarriers can be modulated by adjusting the molecular weight of the grafted polymer and further highlights that functionalized MSN (PEI 10) had better cytocompatibility compared to functionalized MSN (PEI 25). Based on these findings, we chose functionalized MSN (PEI 10) for further experiments.

**Fig. 8:**
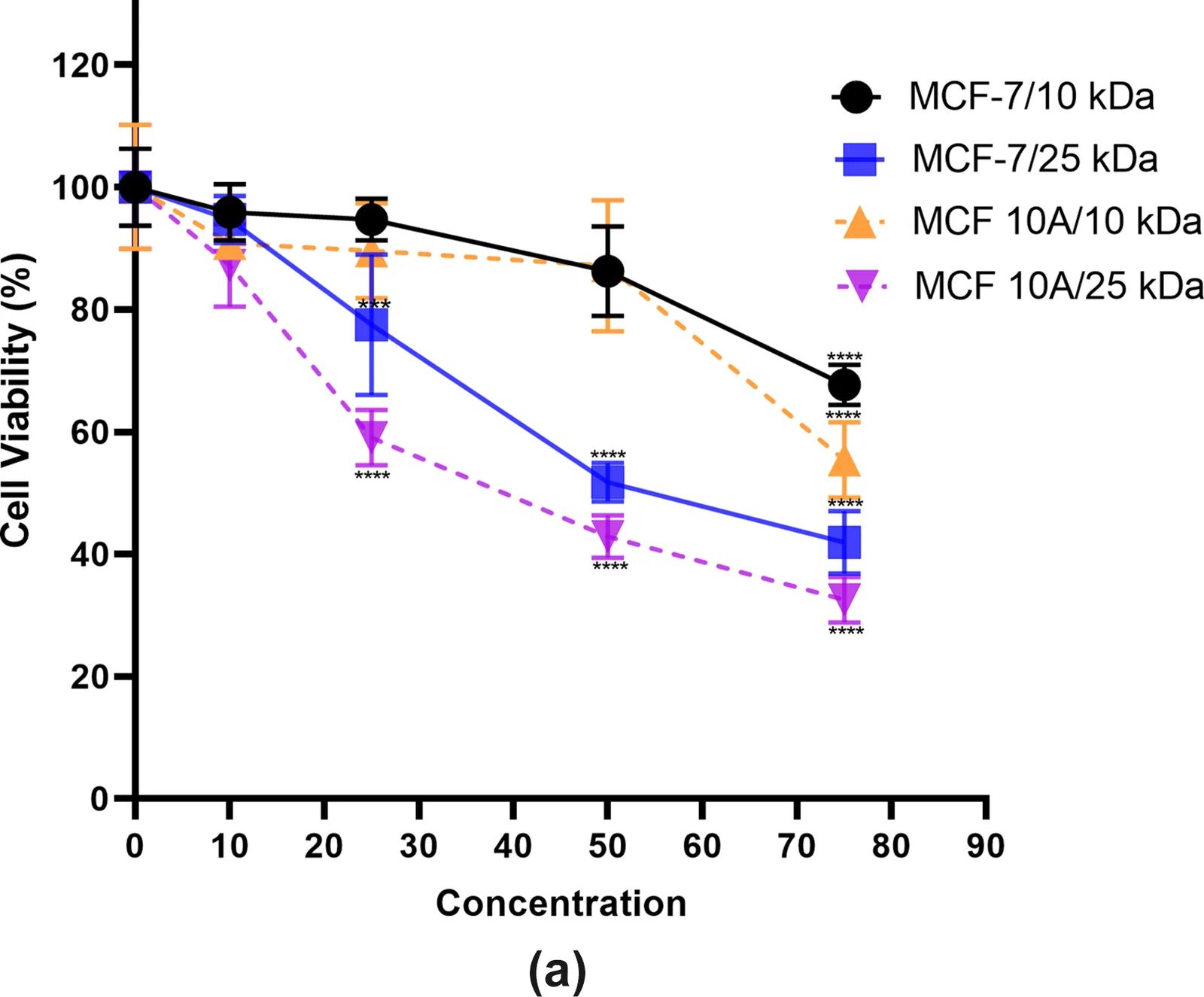

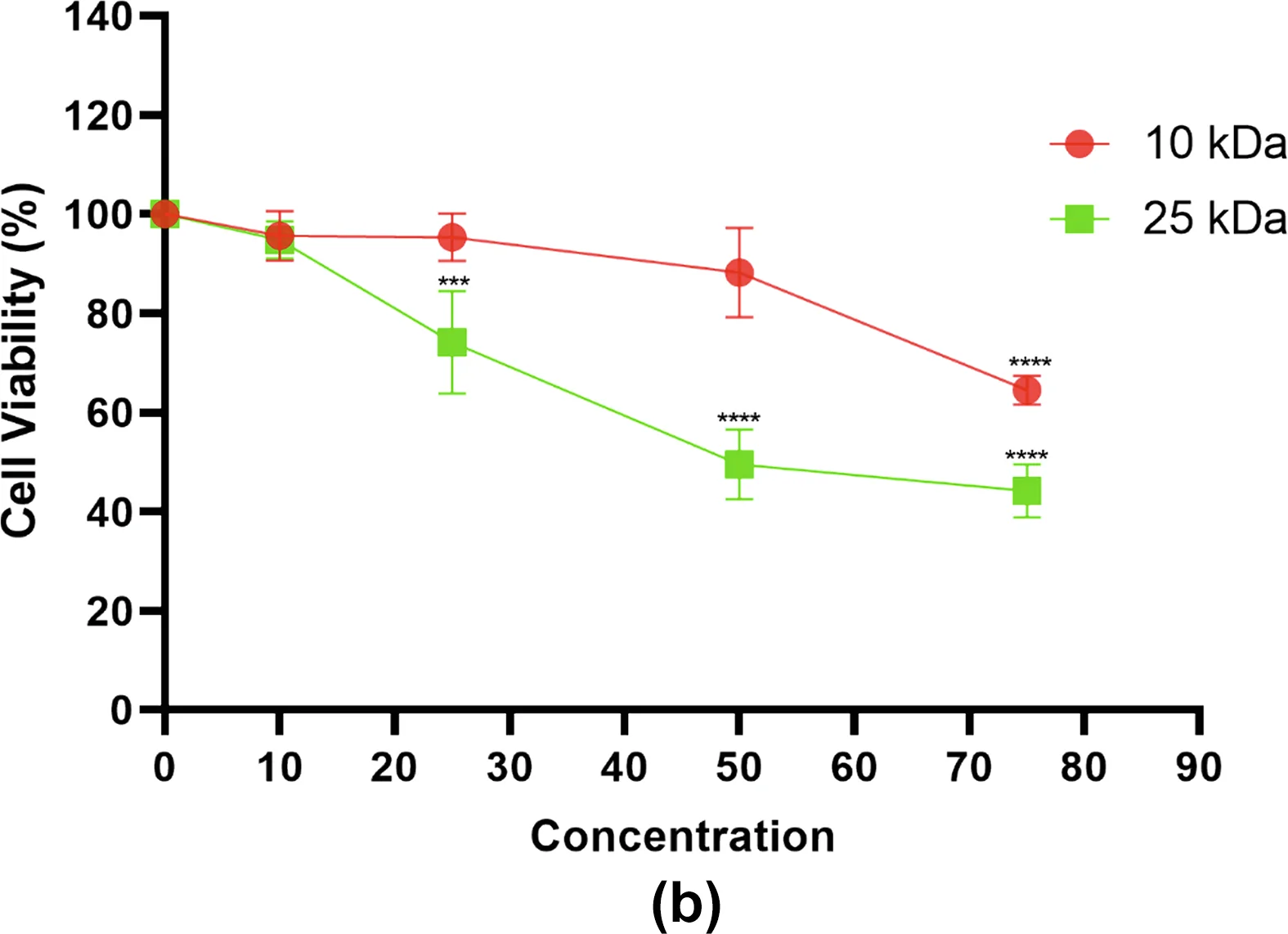
(a) Cell viability assay of fabricated nanocarriers coated with PEI of different molecular weights (10 kDa and 25 kDa) at various concentrations (10, 25,50 and 75 μg mL^-1^) in MCF-7 and MCF 10 A cells. ***p < 0.001 and ****p < 0.0001 compared to their corresponding controls (*n* = 3). (b) Cell viability assay of fabricated nanocarriers coated with PEI of different molecular weights (10 kDa and 25 kDa) at various concentrations (10, 25,50 and 75 μg mL^-1^) in MDA-MB 231. ***p < 0.001 and ****p < 0.0001 compared to their corresponding controls (*n* = 3).

### 3.9 Hemolysis rates

Acceptable blood compatibility of engineered nanocarriers is crucial factor for their potential clinical applications.^43^, ^44^ The absence of detectable hemolysis is generally regarded as an indicator of biocompatibility. In this regard a hemolysis assay was performed to evaluate the hemocompatibility of our nanocarriers. Since damage of RBCs can lead to the release of hemoglobin, the hemolysis rate was quantified by measuring hemoglobin absorbance. Fig. 9 shows negligible hemolysis rates at concentrations of 10, 25 and 50 μg mL^−1^ for the tested nanoparticles. These results indicated our engineered nanocarriers exhibited hemolysis rates less than permissible threshold^45^, ^46^ confirming their good hemocompatibility.

**Fig. 9:**
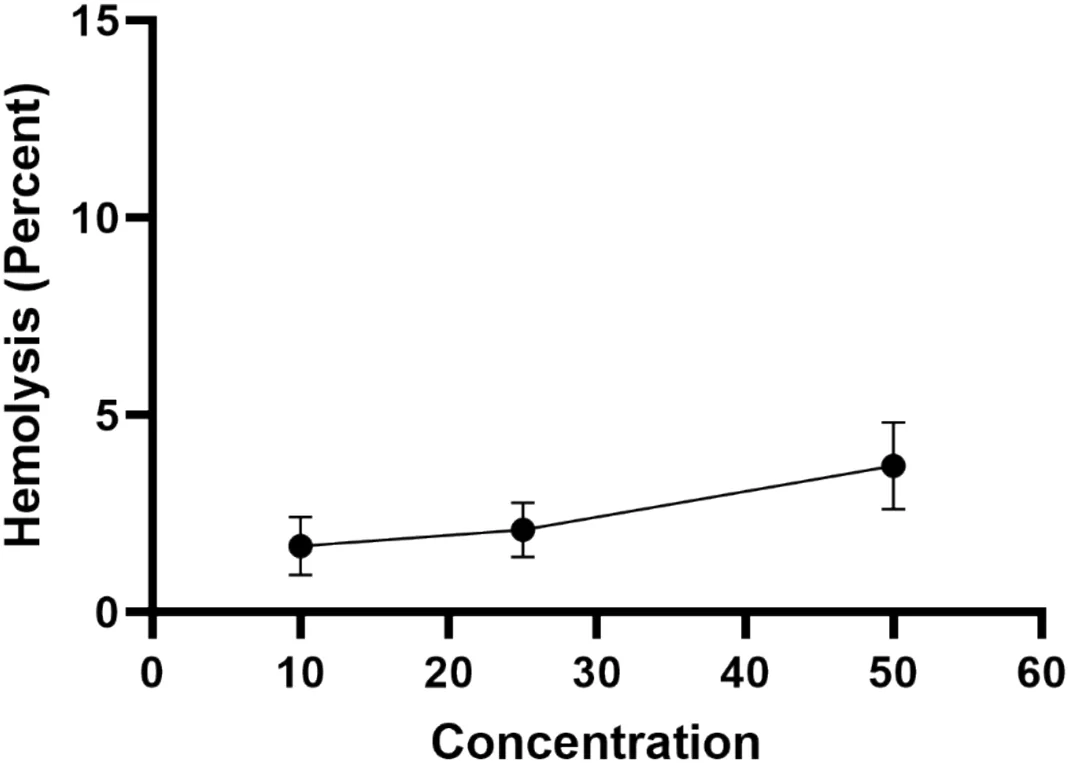
Hemolysis assay of functionalized MSN (PEI 10 kDa) at different concentrations (10, 25,50 and 75 μg mL^-1^). Data are given as the means ± SD (*n* = 3).

### 3.10 Flow cytometric analysis of folate receptor-mediated cell association

Flow cytometric analysis was performed to evaluate the cell association of FITC-conjugated nanoparticles (MSN(FITC)@PEI 10 kDa/FA) in MCF-7 cells. We showed that the incubation of cells with MSN(FITC)@PEI 10 kDa/FA resulted in a significantly higher percentage of FITC-positive cells compared to untreated control, confirming cell association of the nanoparticles. While competition with free folic reduced the percentage of FITC-positive cells confirming folate receptor-mediated binding (Fig. 10).

**Fig. 10:**
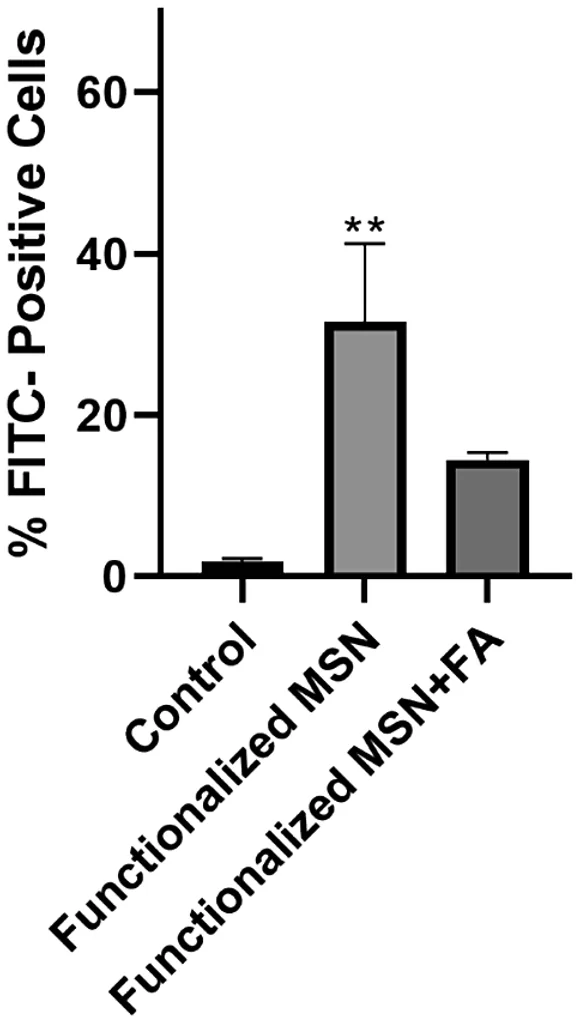
Flow cytometric analysis of FITC conjugated nanocarriers. Functionalized MSN: MCF-7 cells were incubated with functionalized MSN (PEI 10). Functionalized MSN + FA: MCF-7 cells were incubated with functionalized MSN (PEI 10) in the presence of folic acid. **p < 0.01 compared to control. Data are given as the means ± SD (*n* = 3).

### 3.11 mRNA levels analysis

We studied the ability of the siNPT2b complexed with functionalized MSN (PEI 10) to silence the targeted mRNA expression with reverse transcription-polymerase chain reaction (RT-PCR). As shown in Fig. 11a. MCF-7 cells transfected with siNPT2b complexes showed a clear reduction in expression level of NPT2b compared to the cells transfected with NC-siRNA complexes or non-treated cells. Furthermore, quantitative real-time PCR (qRT-PCR) analysis further confirmed these findings and demonstrated a similar pattern of NPT2b reduction (Fig. 11b).

**Fig. 11:**
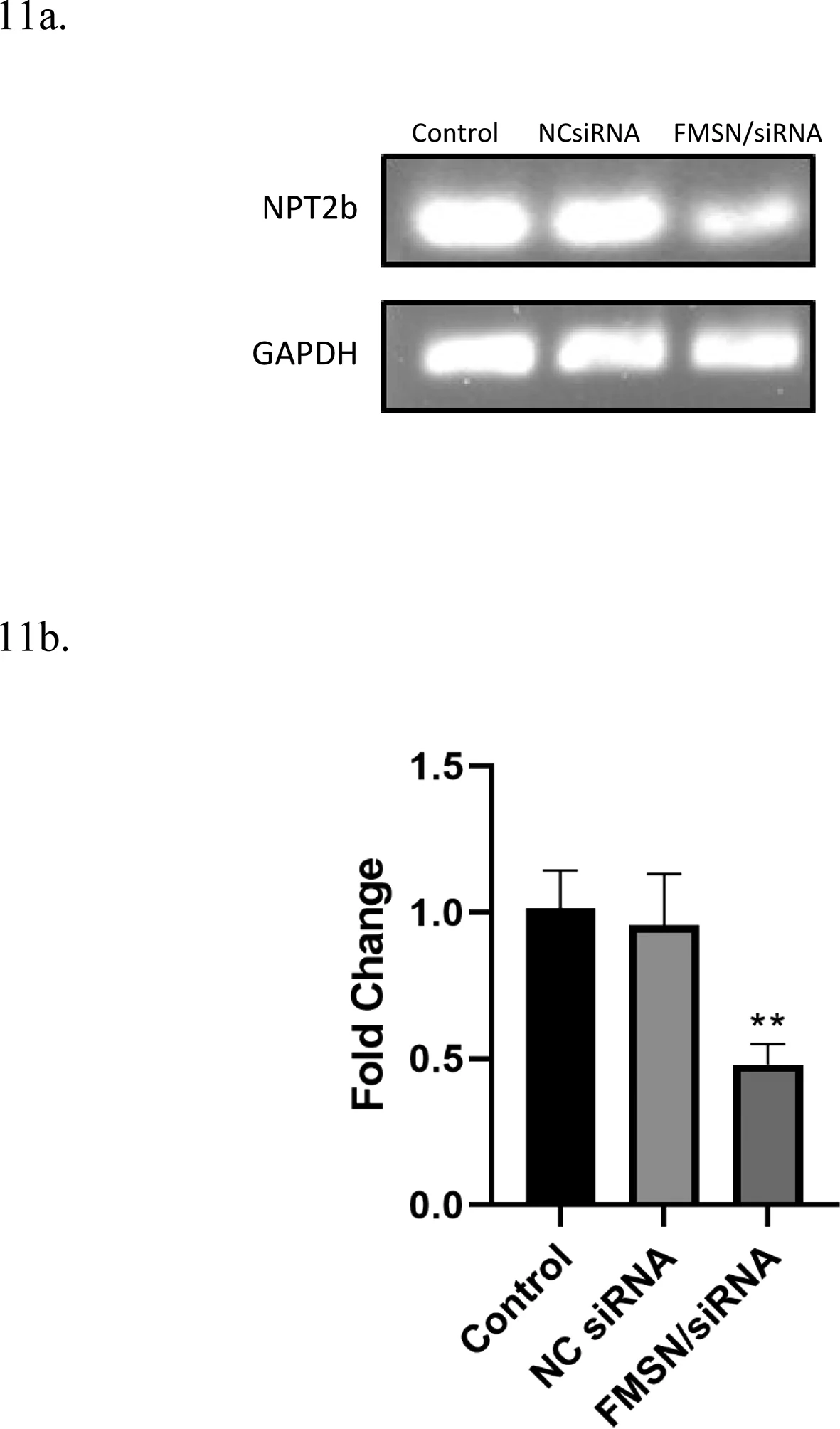
(a) RT-PCR analysis of transfected MCF-7 cells. NC siRNA: Negative Control siRNA, FMSN/siRNA: The complex of functionalized MSN (PEI 10) /siRNA against NPT2b with weight ratio 25. (b) qRT-PCR analysis of transfected MCF-7 cells. NC siRNA: Negative Control siRNA, FMSN/siRNA: The complex of functionalized MSN (PEI 10) /siRNA against NPT2b with weight ratio 25. **p < 0.01 compared to control. Data are given as the means ± SD (*n* = 3).

### 3.12 Cell viability evaluation following transfection

To examine whether efficient knockdown of NPT2b mRNA could regulate cell viability, transfected MCF-7 (Fig. 12a) and MDA-MB231 (Fig. 12b) cells were evaluated using an MTT assay. Our results clearly showed that the cells transfected with siNPT2b complexes exhibited a decreased viability compared to those transfected with NC-siRNA complexes or non-treated cells.

**Fig. 12:**
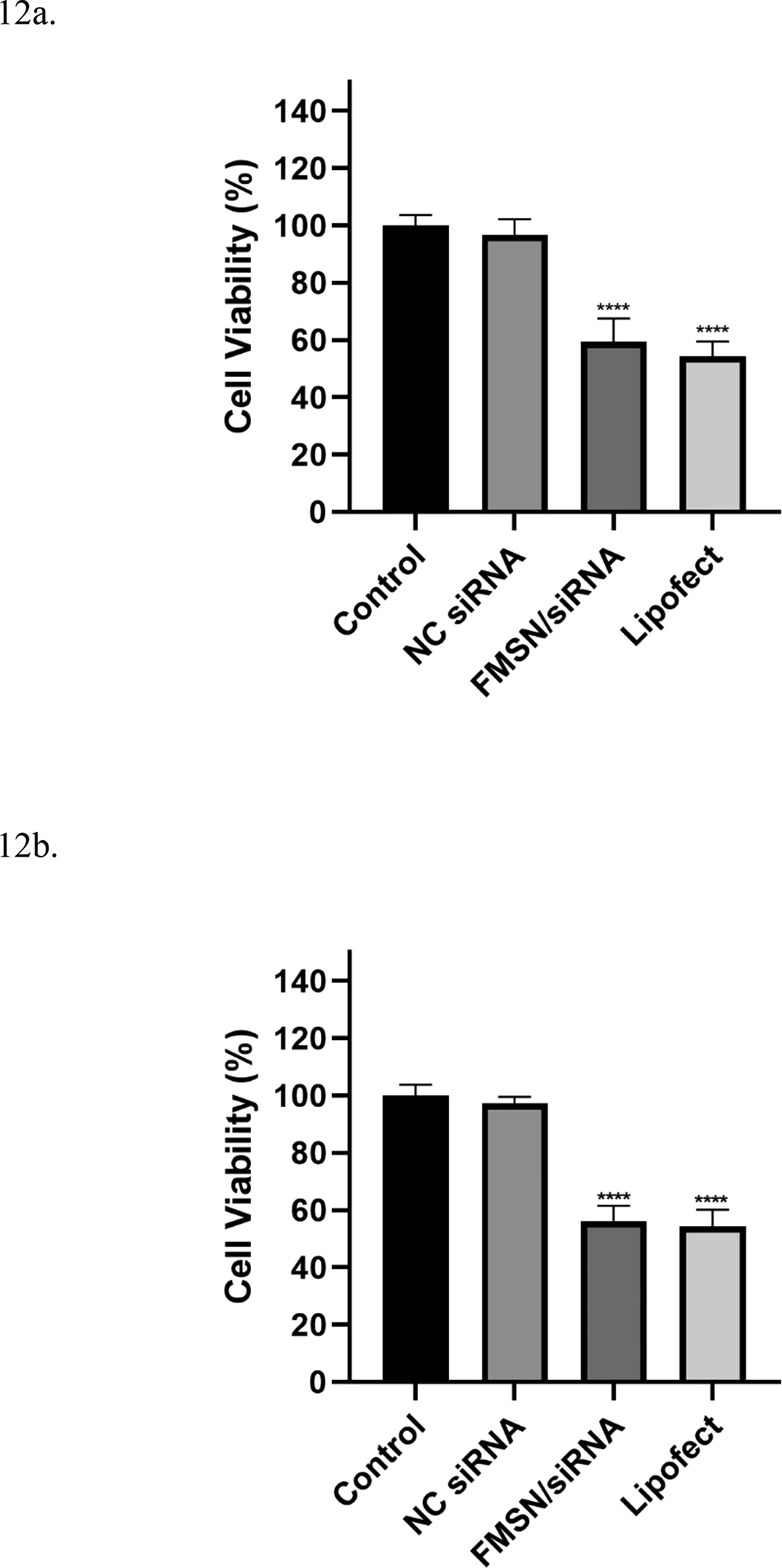
(a) Cell viability assay of fabricated nanocomplexes in MCF-7 cells. NC siRNA: Negative Control siRNA, FMSN/siRNA: The complex of functionalized MSN (PEI 10) /siRNA against NPT2b with weight ratio 25. (b) Cell viability assay of fabricated nanocomplexes in MDA-MB 231 cells, NC siRNA: Negative Control siRNA, FMSN/siRNA: The complex of functionalized MSN (PEI 10) /siRNA against NPT2b with weight ratio 25. ****p < 0.0001 compared to control. Data are given as the means ± SD (*n* = 3).

### 3.13 Apoptosis evaluation

To investigate whether efficient knockdown of NPT2b mRNA could regulate cell death, apoptosis was evaluated in transfected MCF-7 cells using Annexin V-FITC/PI staining followed by flow cytometry analysis. Our results clearly showed that cells treated with the siNPT2b complexes exhibited an increased proportion of apoptotic cells, with a shift from the viable cell population toward early and late apoptotic populations compared with cells treated with NC-siRNA complexes or non-treated cells. These findings suggest that NPT2b knockdown can induce apoptosis in MCF-7 cells (Fig. 13).

**Fig. 13:**
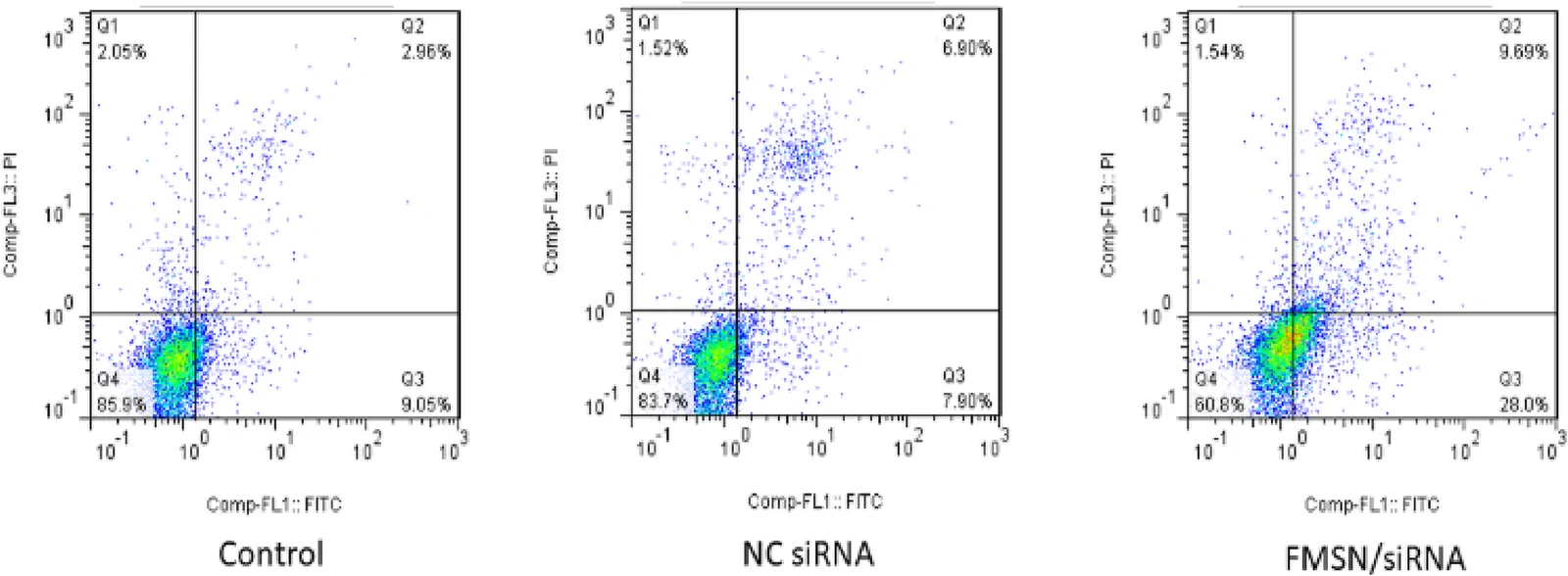
Flow cytometric analysis of apoptosis in MCF-7. NC siRNA: Negative Control siRNA, FMSN/siRNA: The complex of functionalized MSN (PEI 10) /siRNA against NPT2b with weight ratio 25.

## 4 Discussion

MSNs are developing as promising multifunctional platforms for the effective delivery of different agents.^47^, ^48^ MSNs offer various advantages such as customizable surfaces that can be chemically modified to improve carrying a variety of cargos and achieve precise targeting. Their surfaces can be modified for enhanced delivery efficiency by incorporating numerous small molecules such as targeting ligands. Folic acid has been reported to show a selective affinity for folate receptors, which are overexpressed on a variety of cancer cells such as breast cancer cells allowing for targeted delivery while minimizing toxicity to normal cells. By functionalizing MSNs with FA, the delivery of therapeutic agents can be oriented to concentrate at tumor sites, thereby improving treatment effectiveness and reducing side effects.^27^, ^49^, ^50^, ^51^, ^52^ On the other hand, Polyethyleneimine (PEI), a positively charged polymer, is often used to increase the cellular uptake of nucleic acids like siRNA when attached to MSN. The proton sponge effect of PEI promotes endosomal escape, permitting siRNA to effectively enter the cytosol.^53^, ^54^, ^55^ Therefore, in this study, we developed a type of MSN-based system for oligonucleotide delivery, which was grafted with PEI and functionalized by folic acid. Following the synthesis of MSN nanoparticles, we demonstrated that our fabricated MSN nanoparticles exhibited a well-dispersed, spherical morphology with a uniform size distribution with the average particle diameter of approximately 81.9 nm having a highly ordered mesoporous structure within the MSNs. (Fig. 3). Furthermore, to enable nanoparticle tracking, FITC-APTES conjugates were prepared through a nucleophilic addition reaction between isothiocyanate moiety of FITC and the nucleophilic primary amine group of APTES. The resulting FITC–APTES conjugates were subsequently reacted with the hydroxyl groups on the surface of the synthesized MSNs. This conjugation strategy permits the covalent attachment of FITC molecules to APTES, allowing the efficient co-encapsulation of a high density of fluorescent dye molecules inside the porous structure of MSN, thus enhancing their fluorescence loading capacity and stability. Here, we modified the MSN-FITC core surface by adding PEI and the targeting molecule FA as schematically illustrated in Fig. 1, Fig. 2. The emission peak at 469 nm and fluorescence microscopy images clearly confirmed effective surface coverage of the nanoparticles by FITC molecules, exhibiting distinct florescence, and indicated that subsequent PEI-FA modification on FITC-preconjugated MSNs did not distinctly alter their fluorescence intensity (Fig. 4).

On the other hand, the N₂ sorption results demonstrated that the stepwise incorporation of FITC and PEI substantially could alter the textural properties of the MSN, as reflected by the progressive reduction in accessible surface area and pore volume, while preserving its overall mesoporous character. These finding clearly indicated the successful incorporation of the functional components into the MSN structure (Fig. 5 and Table 1).

In addition, FTIR spectroscopy clearly verified the chemical structure of the nanoparticles. The spectra of pure SiO₂ nanoparticles along with those of the surface-modified MSNs (10 or 25 kDa) confirmed the successful incorporation of the intended functional groups, thus validating the rational design and fabrication strategy (Fig. 6).

It has been shown that positively charged delivery systems may have higher cellular internalization in comparison with neutral or negatively charged counterparts.^56^, ^57^ Furthermore, nanoparticles possessing a net positive (cationic) charge have been reported to be particularly suitable in various biomedical applications, including siRNA condensation, drug loading, and the delivery of diagnostic or therapeutic probes.^24^ Our PEI-coated particles exhibit a high positive zeta potential value indicating successful attachment of PEI-FA while suggesting that modified MSN nanoparticles could bind to the negatively charged siRNA through electrostatic interaction. Efficient gene delivery contributes to the ability of carrier to condense nucleic acids into stable nanoparticles, as this protects them from degradation.^58^, ^59^ In this context, the positively charged MSN interact electrostatically with negatively charged siRNA, thus limiting its migration under an electric field.^60^, ^61^ Such condensed complexes have been shown to shield nucleic acids from serum and intracellular nucleases, eventually contributing to better transfection efficiency^62^.

Our result clearly showed that modified MSN successfully protected siRNA from RNase degradation and able to release siRNA molecules almost intact from the complexes following treatment indicating that modified MSNs could protect siRNA from degradation and enable efficient cargo release in target cells (Fig. 7). The toxicity of gene carriers has always been discussed, the cationic nature of PEI can pose toxicity concerns, strategies such as attaching shorter polymer lengths can lighten these effects while maintaining effective delivery capabilities.^56^, ^59^, ^63^, ^64^, ^65^ We addressed this concern by evaluating PEI variants with different molecular weights. In this study, the lower molecular weight PEI (10 kDa) was selected for further experiments due to its lower cytotoxicity compared with PEI 25 kDa, particularly within the concentration range of 1–50 μg mL^-1^ (Fig. 8).

Recent studies have reported that surface-modified MSNs exhibit minimal cytotoxicity and excellent biocompatibility, along with precise targeting capabilities when applied to mammalian cells.^66^, ^67^, ^68^ In line with these reports, our finding clearly confirmed that our fabricated nanocarrier was biocompatible and exhibited efficient cellular binding as determined by hemolysis assay and flow cytometric analysis (Fig. 9, Fig. 10).

It has been reported that inorganic phosphate is required for ATP production, nucleic acid synthesis and phosphorylation-dependent signaling, all of which are upregulated in rapidly proliferating cancer cells.^9^, ^69^, ^70^ To meet this demand, many cancer cells increase phosphate uptake via specific sodium-dependent phosphate co-transporters, including NPT2b. Notably, Altered NPT2b expression has been shown to promote cancer cell proliferation and progression.^71^, ^72^, ^73^ Although its regulation and association with clinical outcomes in breast cancer remains largely uncharacterized, dysregulated phosphate transport may similarly contribute to breast cancer metabolism and progression. Mechanistically, our findings showed that silencing NPT2b using our functionalized nanocarrier [MSN-PEI (10 kDa)-FA] significantly inhibits breast cancer cell viability and induced apoptosis. These effects may occure through three distinct biochemical mechanisms: 1-Bioenergetic stress: downregulating NPT2b limits Pi uptake across the cell membrane, depriving cancer cells of the substrate essential for ATP production during glycolysis and oxidative phosphorylation, thus contributing to an energetic crisis. 2-Attenuation of mitogenic pathways: intracellular Pi levels directly influence pro-survival cascades, notably the Akt/mTOR and MAPK/ERK signaling pathways. Knockdown of NPT2b affects these phosphorylation cascades, downstream protein translation, and cell growth (Hong et al., 2013). 3-Anabolic restriction: Depletion of intracellular Pi stores may limit nucleotide synthesis and phospholipid synthesis needed for membrane duplication during cell division, eventually facilitating cell cycle arrest and apoptosis.^30^, ^70^, ^74^

Therefore, this study aimed to determine whether suppressing NPT2b expression could disrupt phosphate homeostasis and thereby inhibit breast cancer progression, potentially offering a therapeutic strategy. Our results clearly demonstrated that siNPT2B delivery via PEI (10 kDa)-modified MSNs effectively downregulated NPT2b expression, possibly through regulation of phosphate consumption, subsequently leading to the suppression of cancer cell proliferation and induction of apoptosis (Fig. 11, Fig. 12, Fig. 13). Together, these mechanisms explain how nanocarrier-delivered siNPT2b suppresses breast cancer cell growth and support NPT2b as a potential therapeutic.

The MSN-PEI(10 kDa)-FA platform combines the structural stability of inorganic silica, low-toxicity siRNA condensation via optimized 10 kDa PEI, and active folate-receptor targeting. This design overcomes key limitations of liposomal vectors (instability, burst release), high-MW PEI (cytotoxicity), and metallic nanoparticles (low loading capacity). Functionally, the platform permitted safe and selective NPT2b knockdown, regulating phosphate homeostasis and reducing breast cancer cell viability. These results suggest NPT2b as a targetable metabolic vulnerability and position this nanoplatform as a potential nonviral strategy for breast cancer therapy. On the other hand, while these findings highlight the feasibility of NPT2b targeting as a potential therapeutic strategy in phosphate-dependent oncogenic pathways and our system represents a potential proof-of-concept platform for targeted nanotherapeutics. Nevertheless, the current study is limited to in vitro evaluation in breast cancer cell lines. Further investigations are needed to validate the underlying mechanisms, perform detailed bioenergetic profiling, and evaluate the translational potential of this nanomedicine platform through comprehensive in vivo studies, including pharmacokinetic evaluation, biodistribution analysis, and safety assessment in tumor-bearing animal models. These future studies are important to establish the clinical relevance and therapeutic applicability of NPT2b-targeted therapy.

## Declaration of AI use

The authors used AI tools for the purpose of improving English language clarity, grammar, and readability. All AI-suggested changes were critically reviewed and manually adjusted by the authors.

## CRediT authorship contribution statement

**Fatemeh Yazdi Samadi:** Formal analysis, Methodology, Data curation. **Morteza Farab:** Methodology, Data curation. **Mahdieh Emadi:** Methodology, Data curation. **Marzieh Rad Niknam:** Methodology, Data curation. **Maryam Yousefi:** Writing – review & editing, Writing – original draft, Supervision, Methodology, Investigation, Formal analysis, Data curation. **Arash Minai-Tehrani:** Writing – original draft, Project administration, Methodology, Investigation, Formal analysis, Data curation.

## Declaration of competing interest

The authors declare that they have no known competing financial interests or personal relationships that could have appeared to influence the work reported in this paper.
